# Physician-Peer Relationships and Patient Experiences With Specialist Care

**DOI:** 10.1001/jamainternmed.2022.6007

**Published:** 2023-01-03

**Authors:** Maximilian J. Pany, J. Michael McWilliams

**Affiliations:** 1 Department of Health Care Policy, Harvard Medical School, Boston, Massachusetts; 2 Harvard Business School, Boston, Massachusetts; 3 Division of General Internal Medicine and Primary Care, Department of Medicine, Brigham and Women’s Hospital, Harvard Medical School, Boston, Massachusetts; 4 Associate Editor, *JAMA Internal Medicine*

## Abstract

**Importance:**

Peer relationships may motivate physicians to aspire to high professional standards but have not been a major focus of quality improvement efforts.

**Objective:**

To determine whether peer relationships between primary care physicians (PCPs) and specialists formed during training motivate improved specialist care for patients.

**Design, Setting, and Participants:**

In this quasi-experimental study, difference-in-differences analysis was used to estimate differences in experiences with specialist care reported by patients of the same PCP for specialists who did vs did not co-train with the PCP, controlling for any differences in patient ratings of the same specialists in the absence of co-training ties. Specialist visits resulting from PCP referrals from 2016 to 2019 in a large health system were analyzed, including a subset of undirected referrals in which PCPs did not specify a specialist. Data were collected from January 2016 to December 2019 and analyzed from March 2020 to October 2022.

**Exposure:**

The exposure was PCP-specialist overlap in training (medical school or postgraduate medical) at the same institution for at least 1 year (co-training).

**Main Outcomes and Measures:**

Composite patient experience rating of specialist care constructed from Press Ganey’s Medical Practice Survey.

**Results:**

Of 9920 specialist visits for 8655 patients (62.9% female; mean age, 57.4 years) with 502 specialists in 13 specialties, 3.1% (306) involved PCP-specialist dyads with a co-training tie. Co-training ties between PCPs and specialists were associated with a 9.0 percentage point higher adjusted composite patient rating of specialist care (95% CI, 5.6-12.4 percentage points; *P* < .001), analogous to improvement from the median to the 91st percentile of specialist performance. This association was stronger for PCP-specialist dyads with full temporal overlap in training (same class or cohort) and consistently strong for 9 of 10 patient experience items, including clarity of communication and engagement in shared decision-making. In secondary analyses of objective markers of altered specialist practice in an expanded sample of visits not limited by the availability of patient experience data, co-training was associated with changes in medication prescribing, suggesting behavioral changes beyond interpersonal communication. Patient characteristics varied minimally by co-training status of PCP-specialist dyads. Results were similar in analyses restricted to undirected referrals (in which PCPs did not specify a specialist). Concordance between PCPs and specialists in physician age, sex, medical school graduation year, and training institution (without requiring temporal overlap) was not associated with better care experiences.

**Conclusions and Relevance:**

In this quasi-experimental study, PCP-specialist co-training elicited changes in specialist care that substantially improved patient experiences, suggesting potential gains from strategies encouraging the formation of stronger physician-peer relationships.

## Key Points

**Question:**

Do physician-peer relationships motivate improved specialist care for patients?

**Findings:**

In this study of specialty referrals for 8655 patients, patients reported substantially better specialist care than other patients of the same primary care physician (PCP) referred to the same specialty if the consulting specialist trained with the PCP—an association not expected from differences in performance between the same specialists in the absence of PCP-specialist co-training ties. Co-training was not only associated with a more friendly and concerned manner but also clearer explanations, greater engagement in shared decision-making, and changes in prescribing by specialists.

**Meaning:**

This study suggests potentially large gains in quality from encouraging and harnessing physician-peer relationships.

## Teaser

This quasi-experimental study determines whether peer relationships between primary care physicians and specialists formed during training motivate improved specialist care for patients.

## Introduction

It is well understood that physician behavior is a primary driver of patient outcomes and health care spending. Physicians are expected to be well-informed agents who are intrinsically motivated to optimize care for their patients.^1^ Yet deficits in quality are pervasive,^2^ and effective strategies to influence physician decisions and performance remain elusive. That physicians are also motivated by profit^1^ has inspired 2 decades of efforts to link payment to performance on quality measures. These pay-for-performance schemes have produced minimal gains for various reasons, including measurement challenges and inherently weak incentives, and have had unintended consequences.^7^

More recently, interventions have focused on nonfinancial incentives that influence physician behavior. The application of behavioral science to health care^17^ has conceived a range of nudges (eg, use of defaults, public commitments, or information framing) that have successfully prompted higher-value decisions.^19^ While a clear advance, these approaches have so far been applied mainly to specific measures or clinical decisions using scripted interventions, such as peer performance comparisons or required justification to discourage inappropriate antibiotic prescribing.^22^ Broader applications could have a greater impact.

In particular, the influence of peer observation and approval is likely to be powerful in medicine and could be more productively deployed. Physicians are motivated to demonstrate their competence to other physicians not only for financial gain (eg, to earn favorable evaluations as trainees or to attract referrals), but also because they may derive professional satisfaction from upholding standards when observed.

Behavioral science has long demonstrated peer^23^ and audience^26^ effects, by which the physical or imagined presence of others improves performance. These effects can be particularly powerful when peer relationships are strong and when peers share high standards and common purpose.^27^ Accordingly, an audience of familiar peers may elevate physician performance, not only by subjecting it to informed scrutiny, but also by providing an opportunity to demonstrate commitment to what is valued by the profession. If strong, the motivational effects of peer interaction^25^ could have profound implications for the organization of care delivery, including potential gains from models encouraging peer familiarity and visibility—gains that could accrue over many dimensions of care without requiring decision-specific interventions.

In this study, we used 2016 to 2019 electronic health record data on referrals initiated by primary care physicians (PCPs) from a large academic health system to investigate the association between PCP-specialist co-training and patient experiences with specialist care. Training together is 1 source of professional peer relationships that, when present, may motivate physicians to deliver better care. Specialists are aware that PCPs can observe aspects of their care through reading their notes and talking to patients; the presence of a strong peer relationship may also remind them of commonly valued precepts of professionalism. Accordingly, specialists may aspire to deliver their best care when seeing patients whose PCPs they know.

## Methods

### Study Design and Overview

In a difference-in-differences analysis of specialist visits resulting from referrals, we compared patient ratings of specialist care between patients seen by a specialist who trained with the patient’s PCP (in medical school or postgraduate medical programs) and patients of the same PCP seen by a specialist who did not train with the PCP, controlling for any between-specialist differences in ratings observed in the absence of co-training ties between PCP-specialist dyads. The study was approved by the health system’s institutional review board. Informed consent was not required because of the nature of the research as analyzing secondary data. Data were collected from January 2016 to December 2019 and analyzed from March 2020 to October 2022.

The referral ordering system generates 2 types of referrals: directed referrals, in which PCPs specify a specialist by name in the referral order, and undirected referrals, in which PCPs do not. Undirected referrals are assigned to specialists within a specialty department based on specialist availability and patient input.

We constructed 2 analytic samples for the analysis of the study’s primary outcome. First, we pooled directed and undirected referrals to maximize available information on patient ratings of specialist visits and number of PCP-specialist dyads who trained together. Second, we limited analysis to the set of undirected referrals. These should be less subject than directed referrals to potential bias from PCPs referring certain patients selectively to specialists with whom they trained (eg, in an attempt to improve match quality^29^) or from PCPs priming patient expectations when referring to a specialist they know well.

Assignment of undirected referrals may be influenced by patient preferences based, for example, on recommendations from friends or family, patient availability (patients with less availability may prefer specialists with greater availability), specialists’ public reputation, or PCP recommendations not reflected in the referral order. However, we controlled for each specialist’s average effect (pooled across PCPs) in addition to each PCP’s average effect to isolate the relationship between PCP-specialist dyad *concordance* in training and patient experiences with specialist care. Thus, if a specialist with whom a PCP trained was rated highly because they provided better care or selectively attracted patients who gave favorable ratings, our analytic approach would remove that specialist’s higher average rating from the estimated effect of co-training ties. We assumed only that any nonrandom sorting of patients to specialists did not vary systematically across PCPs according to PCP-specialist co-training ties. This assumption would be violated in undirected referrals if, for example, PCPs verbally instructed certain types of patients to request a specialist with whom they trained. We tested this assumption by assessing the relationship between PCP-specialist co-training and patient characteristics.

### Study Population

We identified all referrals initiated by system-affiliated PCPs from 2016 to 2019 to 13 medical and surgical specialties accounting for 50.7% of total system referrals (eMethods in Supplement 1). We limited referrals to those that resulted in a completed specialist consultation visit (linked to the referral order by a tracking identifier) and were thus eligible for patient experience assessment. We further restricted the study analysis to patients new to the specialist (eMethods in Supplement 1) because referrals for established patients may have been ordered to continue a preexisting relationship. We also restricted analyses to visits with specialists who trained with at least 1 PCP in their referral base and could thus contribute to co-training estimates. We defined a specialist’s referral base as all PCPs belonging to the physician organizations that included at least 1 PCP who referred to the specialist. Finally, for analyses of our primary outcome (patient experiences), we included only specialist practices affiliated with the 2 hospitals in the system (1 large academic and 1 community) that routinely administered patient experience surveys.

### Study Variables

#### Co-Training

Using medical licensing data from the Massachusetts Board of Registration in Medicine, we determined whether each referring PCP and consulting specialist overlapped at the same institution for at least 1 year during either medical school or postgraduate medical training irrespective of stage (ie, residency or fellowship) (eMethods in Supplement 1). Because this indicator of co-training is a predictor of peer relationships, not a direct measure, the study results are likely biased toward the null. In sensitivity analyses, we explored whether results differed for PCP-specialist dyads with full temporal overlap (those who were in the same medical school class or post-graduate training cohort and therefore more likely to form relationships) vs partial overlap and for dyads who overlapped in medical school vs post-graduate training.

#### Patient Experiences With Specialist Care

**Figure 1.**
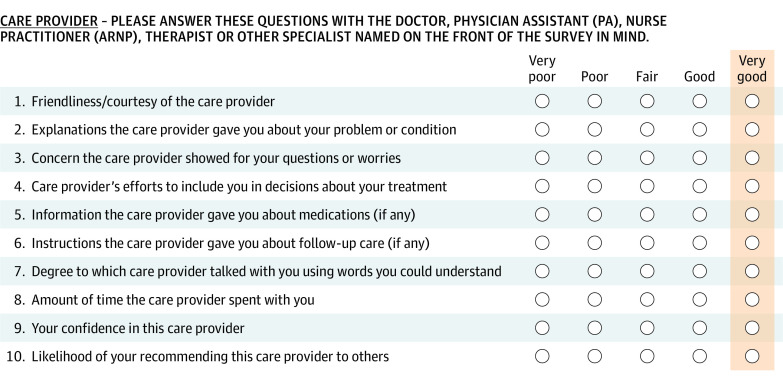
Press Ganey Medical Practice Survey Care Provider Questions Patients rated their specialists on a scale from 1 (very poor) to 5 (very good) on 10 items, as shown in the figure. We constructed a composite rating by first dichotomizing (*very good *vs other) and centering responses and then calculating a mean rating among all nonmissing, centered, dichotomized items for each specialist visit with data available. Press Ganey has granted permission to reproduce this copyrighted figure.

The study’s primary outcome was a composite measure of patient-reported experiences with ambulatory specialist care based on the Care Provider domain of the Press Ganey Medical Practice Survey, which includes 10 questions (Figure 1). The survey was administered via mail and email after a random sample of ambulatory visits at 2 system hospitals during the study period with a reported response rate of 23%.

We focused on patient experiences as our primary outcome for 3 main reasons. First, patient experiences constitute an important dimension of quality, one that is positively associated with other important care processes and outcomes.^31^ Second, patient experience measures should detect physician efforts to demonstrate their professionalism, as the medical profession values patient-centered care. Third, we could attribute patient experience ratings to specific specialist visits because the survey referenced a specific visit and was completed soon after the visit. In contrast, the effects of enhanced specialist care on more distal outcomes (eg, disease control, complications, or mortality) would be challenging to ascertain, as such outcomes manifest with substantial lags and reflect many other care inputs.

We dichotomized responses to each item into *very good* (the highest rating) vs other,^34^ thereby facilitating interpretation with estimates expressed in terms of the percentage of patients giving their specialist a top rating. We calculated a composite equal to the average of nonmissing values for the dichotomized item, each centered around its sample mean. In secondary analyses, we also assessed the contribution of each of the 10 survey items to the primary estimate.

### Additional Analyses to Explore Mechanisms

In addition to the sensitivity analyses described above, we assessed whether patient ratings of the same specialist differed between directed and undirected referrals to further explore whether the study’s findings were due to PCPs sharing their impressions of specialists in advance as opposed to specialists responding to the presence of a strong peer connection. If visits following directed referrals were rated systematically higher, consistent with a priming effect, that might raise concern about residual bias from unobserved priming among undirected referrals.

As secondary outcomes, we examined 3 indicators of specialist ordering during the consultation: whether the specialist prescribed a medication, ordered an imaging study, or requested a follow-up appointment be scheduled at check out. We could not assess the appropriateness of these orders, or whether they added to or merely preempted other orders, but they provided an objective basis for assessing changes in specialist behavior other than in interpersonal communication. Because these outcomes could be assessed for all specialist visits in the entire system, not just those for which patient experiences were collected, we focused on undirected referrals in analyses of these secondary outcomes.

### Statistical Analysis

For each outcome, we fit the following linear regression model:
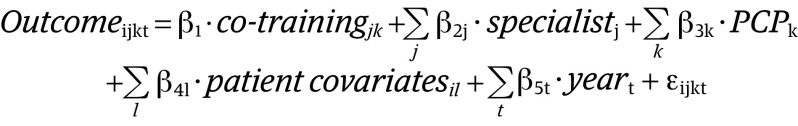
where *i* indexes the completed referral with specialist *j* for a patient of PCP *k* in year* t.* The specialist and PCP fixed effects (the β_2_ and β_3_ terms) control for the average patient rating of specialist care associated with each specialist and PCP, respectively, and thus for any physician-specific factors (eg, specialists’ availability, specialists’ interpersonal skills, or PCPs’ referral thresholds) that might influence patient ratings. With specialist and PCP fixed effects in the model, the coefficient on the indicator of co-training (β_1_) is the average difference in the outcome between patients seen by a specialist who trained with the referring PCP and patients of the same PCP seen by other specialists, controlling for the difference in the outcome between the same specialists for patients within other PCPs (the difference that would be expected in the absence of the dyad’s co-training tie). Assuming the co-training status of the PCP-specialist dyad is independent of patient factors conditional on the referring PCP and consulting specialist (ie, the distribution of referred patients' characteristics across specialists did not vary by PCP according to who the PCP trained with), β_1_ is the average causal effect of a PCP-specialist dyad having overlapped in their training. To assess the validity of this assumption, we estimated this effect with and without adjustment for patient covariates (Table). A minimal effect of adjustment would support the assumption. We used a robust variance estimator,^37^ specifying referring PCPs as clusters. Two-sided statistical significance was assessed at *P* < .05; R statistical software, version 3.6 (The R Project for Statistical Computing), and Python, version 2.7 (Python Software Company), were used.

**Table.** Association Between Co-Training Status of PCP-Specialist Dyad and Characteristics of Patients Seen During Visits With Specialists

Variable	Visits with specialists, mean or %^a^		Standardized mean difference	Effect of single variable adjustment on main outcome^b^
	Co-trained with PCP	Did not co-train with PCP		
Mean age, y (SD)	56.7 (12.1)	57.4 (10.6)	−0.064	7.9
Female	62.1	63.0	−0.027	3.2
Race and ethnicity^c^				
Asian	2.8	2.9	−0.002	3.8
Black	5.4	4.4	0.063	5.4
Hispanic	0.8	0.9	−0.018	3.7
Non-Hispanic, White	86.7	86.7	−0.001	3.8
Other	4.2	5.1	−0.049	2.6
Preferred language				
English	99.5	97.6	0.157	2.6
Spanish	0.2	0.8	−0.083	3.7
Other	0.3	1.6	−0.132	2.6
Educational attainment, highest degree				
High school	18.9	18.8	0.002	3.7
College	50.7	50.9	−0.005	3.8
Graduate school	15.5	16.8	−0.041	4.1
Other	14.9	13.5	0.050	4.4
Insurance				
Commercial	60.7	59.3	0.034	4.1
Medicare	25.3	26.8	−0.042	4.2
Medicaid	5.4	5.3	0.002	3.8
Other	8.6	8.5	0.006	3.8
Mean Elixhauser comorbidity index (SD)^d^	1.73 (2.21)	1.86 (1.82)	−0.070	4.4

Abbreviation: PCP, primary care physician.

^a^ Table shows patient characteristics for the primary sample that includes all referrals (directed and undirected) with patient experience ratings available. Means or proportions in characteristics, and differences associated with co-training, were estimated and adjusted for specialist and PCP fixed effects by fitting a model for each patient covariate as the dependent variable with an indicator of co-training status and specialist and PCP fixed effects as the independent variables. Numbers may not sum to 100% due to rounding.

^b^ The effect of single-variable adjustment on the main outcome (ie, the difference in composite patient experience ratings between visits with co-trainees vs non-co-trainees) denotes the percentage change in the outcome when adjusting for the indicated covariate (but not other covariates) relative to a model adjusting only for specialist and PCP fixed effects.

^c^ Race and ethnicity are assessed from electronic health record data, which may not concord with self-reported race and ethnicity.^35^ As with other patient covariates, we assessed race and ethnicity to assess balance in patient characteristics associated with co-training relationships.

^d^ The Elixhauser comorbidity index summarizes patient comorbidities that are predictive of hospital outcomes, including mortality, and was measured over the 12 months preceding each patient’s visit.^36^

## Results

### Study Sample

The primary analysis of patient experiences included 9920 specialist visits (5562 resulting from directed and 4358 from undirected referrals) for 8655 patients (62.9% female; mean age, 57.4 years) with 502 specialists in 13 specialties (eFigure; eTables 1-2 in Supplement 1). Systemwide, 137 074 specialist visits resulting from undirected referrals were available for analysis of secondary outcomes. As expected, only a small proportion of specialist visits involved a PCP-specialist dyad who overlapped in training (3.1% [306 of 9920] and 3.0% [4104 of 137 074] of visits in the aforementioned samples, respectively).

### Covariate Balance

Among visits resulting from all referrals at the 2 system-affiliated hospitals routinely collecting patient experience data, PCP-specialist co-training ties were not associated with a meaningful difference in the availability of completed patient experience survey data (covariate-adjusted difference: −0.6 percentage points [pp]; 95% CI, −2.6 to 1.3 pp) (eResults in Supplement 1). Among visits from directed or undirected referrals with patient experience data available, patient characteristics differed minimally by co-training status of PCP-specialist dyads (Table). Differences were still modest but subject to more random error in the smaller sample of undirected referrals with patient experience data available and were very small in the larger systemwide sample of undirected referrals (eTables 3-4 in Supplement 1).

### Patient Experiences With Specialist Care

**Figure 2.**
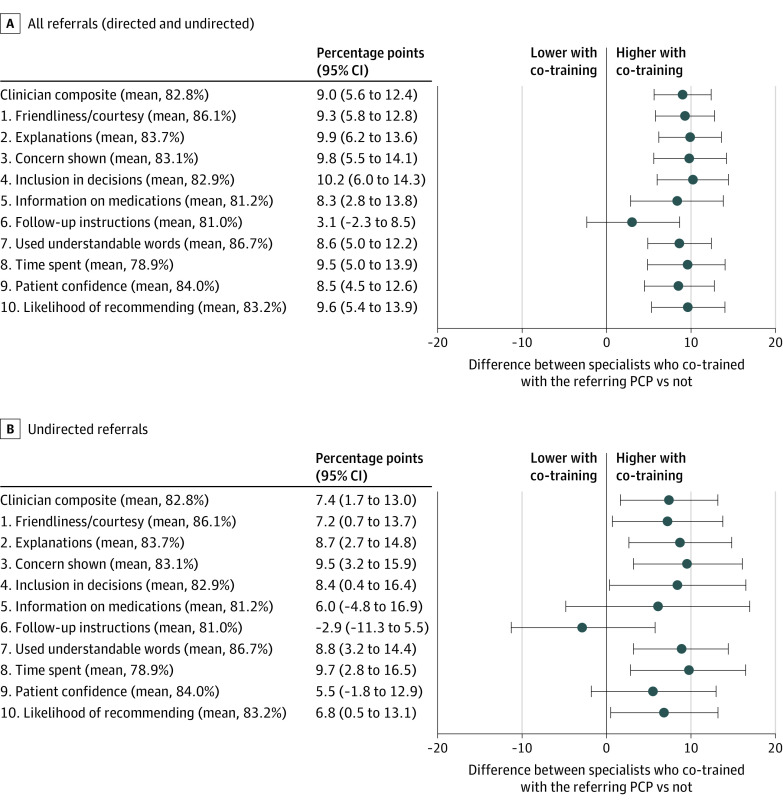
Adjusted Differences in Patient Experience Ratings of Specialist Care Between Visits With Specialists Who Co-Trained With the Referring PCP and Visits With Specialists Who Did Not A, Estimates in the sample of specialist visits resulting from all referrals (directed and undirected). B, Estimates in the sample of specialist visits resulting from undirected referrals only. Point estimates (in percentage points) are plotted with 95% CIs for differences in patient experience ratings between visits with specialists who co-trained with the referring primary care physician (PCP) vs those who did not. The vertical line at 0 denotes no effect.

PCP-specialist co-training was associated with a 8.3 pp higher composite rating of specialist care (95% CI, 4.9-11.8 pp; *P* < .001) when controlling for year and PCPs’ and specialists’ average ratings (fixed effects) but not patient covariates. Adjustment for patient covariates slightly strengthened, rather than attenuated, the association (9.0 pp; 95% CI, 5.6-12.4 pp; *P* < .001) (Figure 2A). This difference corresponds to an effect size of 1.31 SDs of the specialist-level distribution in composite ratings, analogous to an improvement from median performance among specialists to the 91st percentile. Item-specific analyses revealed consistently strong associations between co-training and patient experiences for 9 of 10 survey items (Figure 2A and B).

Estimates were qualitatively similar when we restricted to specialist visits resulting from *undirected* referrals (Figure 2 B; eTable 3 in Supplement 1). Alternate definitions of co-training all produced positive associations between co-training and patient experiences but suggested stronger associations when co-training ties were based on overlapping postgraduate (vs medical school) training and full (vs partial) temporal overlap in training (eTables 5-6 in Supplement 1). Controlling for visit-level medication and imaging orders did not attenuate the association between co-training and patient experiences (eTable 7 in Supplement 1).

### Specialists’ Ordering Behavior

**Figure 3.**
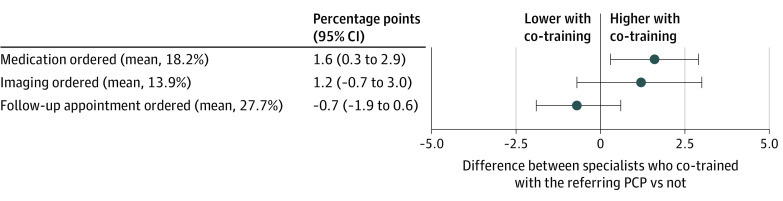
Adjusted Differences in Specialist Orders Between Visits With Specialists Who Co-Trained With the Referring PCP and Visits With Specialists Who Did Not Point estimates (in percentage points) are plotted with 95% CIs for differences in electronic health record–recorded specialist orders between visits with specialists who co-trained with the referring primary care physician (PCP) vs those who did not, in the sample of undirected referrals. The vertical line at 0 denotes no effect.

PCP-specialist co-training was associated with a 1.6 pp (95% CI, 0.3-2.9 pp) higher adjusted proportion of specialist visits in which patients were prescribed a medication (8.8% of the sample mean [18.2%]), a less precisely estimated but similarly large 1.2 pp (95% CI, −0.7 to 3.0 pp) higher adjusted proportion of visits with imaging ordered (8.6% of the sample mean [13.9%]), and no difference in the proportion with a follow-up appointment recommended (Figure 3).

### Sensitivity Analyses

**Figure 4.**
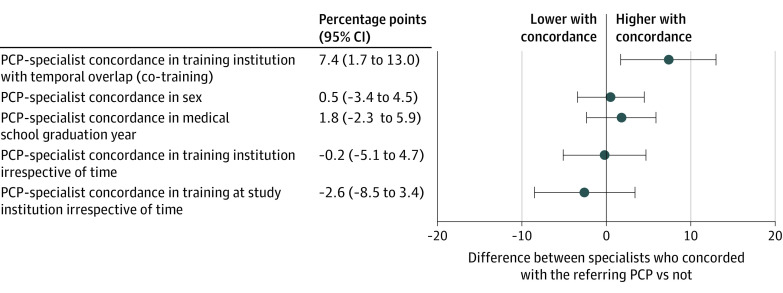
Adjusted Differences in Composite Patient Ratings of Specialist Care Associated With PCP-Specialist Concordance in Select Characteristics Point estimates (in percentage points) are plotted with 95% CIs for differences in composite patient experience ratings between visits with specialists who concorded with the referring primary care physician (PCP) on the indicated measures vs those who did not, in the sample of undirected referrals. The vertical line at 0 denotes no effect. Concordance in medical school graduation year refers to a difference in graduation year of 3 or fewer years, without concordance in training institution. Concordance in training institution denotes PCP-specialist dyads who trained at the same institution (including the study institution but also all other institutions represented in the study sample), while concordance in training at the study institution refers to PCP-specialist dyads who trained at the institution at which the study was conducted.

In contrast with co-training, PCP-specialist concordance in sex, concordance in year of medical school graduation without concordance in training institution, training at the same institution irrespective of temporal overlap, and concordant training at institutions affiliated with the study health system irrespective of temporal overlap were weakly associated with patient ratings of specialist care (Figure 4). The within-specialist difference in patient ratings between directed and undirected referrals was minimal (eTable 8 in Supplement 1).

## Discussion

In this quasi-experimental study of specialist referrals in a large health system, patients’ ratings of specialist care were markedly higher when the specialist and referring PCP trained at the same time at the same institution, particularly when in the same year, and were thus more likely than other PCP-specialist dyads to have a strong peer relationship established. The enhanced patient experiences were not explained by the performance of the same PCPs or specialists in the absence of co-training ties or by concordance in other physician characteristics that, alone, would not be expected to result in strong peer relationships. We also found no evidence of selection bias from nonrandom sorting of patients to specialists with concordant training. These findings suggest that the higher patient ratings of specialists were specific to the PCP-specialist relationship as opposed to PCP, specialist, or patient-specific factors. In addition, patient ratings of specialist care were not higher in directed referrals, in which PCPs selected a preferred specialist for patients and were thus more likely to share their impressions of the consulting specialist with the patient. This suggests that any unobserved disclosure of favorable impressions in undirected referrals are also unlikely to explain the study results, leaving specialist responses to relationships with PCPs as the more likely explanation.

Secondary analyses suggest that referrals from a PCP with whom a specialist overlapped in training not only elicited a more friendly, courteous, and concerned manner but also clearer explanations, greater engagement in shared decision-making, more time spent with patients, and objective changes in medication prescribing. Thus, the behavioral response elicited by co-training ties seems to extend well beyond a change in demeanor that might alter patient perceptions to include behavioral changes that promoted patient-centered care. We could not judge the appropriateness of resulting changes in ordering behavior, which could signify changes in decision-making (eg, from gathering additional information from patients), initiation of treatment plans, courtesy refills, or unnecessary care. Findings for patient experiences were not mediated by changes in medication or imaging ordering.

Taken together, these findings are consistent with the notion that peer relationships can motivate physicians to deliver improved care through *peer* or *audience effects*^23^; when physicians believe their work may be scrutinized or recognized by peers, they may aspire to higher standards. Referrals from familiar PCPs should make alignment of values more visible, thereby establishing stronger peer accountability and offering an opportunity for the specialist to demonstrate what is valued by the related PCP-specialist dyad, including competence and patient-centered care.^25^

The estimated effects of co-training on patient ratings of specialist care are much larger than the effects of other policies, interventions, or efforts to improve patient experiences, including public reporting,^38^ accountable care organizations,^39^ health plan effects,^40^ and hospital characteristics.^41^ Accordingly, the present study’s findings, and extensions thereof, could have major implications for the organization of care delivery, as they suggest potentially large quality gains from models encouraging peer interactions—such as team-based care, digital consultations that foster direct communication, peer coaching, and multispecialty case discussions.

Whereas much of the literature on peer motivation in the workplace focuses on synchronous in-person interactions,^23^ our findings suggest that peer relationships can affect physician behavior when peer interactions are asynchronous and virtual. In addition, the peer relationships in our study were formed in the past (during training) but elicited tangible benefits for patients many years later, suggesting that delivery models that strengthen peer relationships may continue to yield payoffs over time. Peer and audience effects are at play during training and conceivably drive much of the motivation to learn and provide high-quality care but are not cultivated in the routine practice of medicine thereafter, suggesting substantial room for fostering and harnessing peer relationships more systematically. More generally, our study suggests that nonfinancial strategies to harness physician professionalism, including the pressure to live up to the expectations of colleagues, could generate gains over many dimensions of care without requiring decision-specific interventions that risk erosion of intrinsic motivation when deployed in aggregate.^16^

In our study’s setting, patient characteristics were balanced with respect to co-training ties. Across other settings, patients exposed to strong physician-peer relationships may differ systematically from those in settings where physicians have weaker relationships. In addition, some physicians may favor certain patients in referring to specialists well-known to them, plausibly contributing to health care disparities. Nevertheless, the main implication of our findings is that peer relationships could be fostered and deployed to improve quality more broadly and even reduce disparities (eg, by implementing team care models in community health centers).

### Limitations

This study has limitations. First, we investigated PCP-specialist co-training as a proxy for peer relationships that may motivate physicians to deliver better care, but co-training ties were uncommon and likely not the only source of such peer effects. Whether other sources of physician peer relationships produce similar effects remains a question for future work. Nevertheless, the implications of our findings may generalize, as the professional or social relationships formed in training could be replicated to some extent via other strategies that encourage physicians to interact. Thus, although co-training is a special case, it is observable and instructive.

Second, the response rate to the patient experience survey was low, as is characteristic of patient surveys. However, this should not compromise the validity of this study as long as PCP-specialist co-training did not affect survey response; we found no evidence that it did. Third, we could not definitely exclude the possibility that our findings were mediated by affinity bias that induced higher specialist performance as a consequence of shared traits rather than strong relationships. While affinity bias could result from shared affiliation with a training institution, shared affiliations alone, without temporal overlap, were not associated with patient experiences. Only when physicians trained at the same institution at the same time did we find an association. Fourth, the impact of peer relationships formed from co-training observed at the study institution may not generalize to other institutions.

Fifth, we also could not exclude the possibility that differences in unobserved patient characteristics confounded our estimates. Nevertheless, the large magnitude of effects on patient experiences in addition to the observed balance on patient characteristics make this explanation highly unlikely.

## Conclusions

In conclusion, PCP-specialist co-training led to significantly improved patient experiences with specialist care, suggesting the possibility of considerable quality gains from strategies encouraging the formation of stronger peer relationships among physicians.

## Notes

Supplement 1. **eMethods.** Supplemental methods **eResults.** Supplemental results **eFigure.** Conceptual representation of the study design **eTable 1**. Combined sample characteristics by specialty **eTable 2.** Non-response to patient experience items **eTable 3.** Association between co-training status of PCP-specialist dyad and characteristics of patients seen during visits with specialists, in the sample of undirected referrals with patient experience ratings available **eTable 4.** Association between co-training status of PCP-specialist dyad and characteristics of patients seen during visits with specialists, in the sample of system-wide undirected referrals **eTable 5.** Decomposition of the co-training effect by overlap in medical school versus post-graduate medical training **eTable 6.** Decomposition of the co-training effect by full temporal overlap (same-year trainees) versus partial temporal overlap (different-year trainees) **eTable 7.** Association between co-training and composite patient experience with specialist care, controlling for medication and imaging orders **eTable 8.** Association of composite patient experience with specialist care and PCP decisions to direct referrals

Supplement 2. Data sharing Statement
