# Supplementary material for: Physician-Peer Relationships and Patient Experiences With Specialist Care
Source: JAMA Intern Med. 2023 Jan 3:e226007. Online ahead of print. doi: 10.1001/jamainternmed.2022.6007 (PMC9857606; doi:10.1001/jamainternmed.2022.6007)
Supplement: Supplement 1. — eMethods. Supplemental methods eResults. Supplemental results eFigure. Conceptual representation of the study design eTable 1. Combined sample characteristics by specialty eTable 2. Non-response to patient experience items eTable 3. Association between co-training status of PCP-specialist dyad and characteristics of patients seen during visits with specialists, in the sample of undirected referrals with patient experience ratings available eTable 4. Association between co-training status of PCP-specialist dyad and characteristics of patients seen during visits with specialists, in the sample of system-wide undirected referrals eTable 5. Decomposition of the co-training effect by overlap in medical school versus post-graduate medical training eTable 6. Decomposition of the co-training effect by full temporal overlap (same-year trainees) versus partial temporal overlap (different-year trainees) eTable 7. Association between co-training and composite patient experience with specialist care, controlling for medication and imaging orders eTable 8. Association of composite patient experience with specialist care and PCP decisions to direct referrals [file jamainternmed-e226007-s001.pdf]

Supplemental Online Content

Pany MJ, McWilliams JM. Physician-peer relationships and patient experiences with specialist care. *JAMA Intern Med.* Published online January 3, 2023. doi:10.1001/jamainternmed.2022.6007

eMethods. Supplemental methods

eResults. Supplemental results

eFigure. Conceptual representation of the study design

eTable 1. Combined sample characteristics by specialty

eTable 2. Non-response to patient experience items

eTable 3. Association between co-training status of PCP-specialist dyad and characteristics of patients seen during visits with specialists, in the sample of undirected referrals with patient experience ratings available

eTable 4. Association between co-training status of PCP-specialist dyad and characteristics of patients seen during visits with specialists, in the sample of system-wide undirected referrals

eTable 5. Decomposition of the co-training effect by overlap in medical school versus post-graduate medical training

eTable 6. Decomposition of the co-training effect by full temporal overlap (same-year trainees) versus partial temporal overlap (different-year trainees)

eTable 7. Association between co-training and composite patient experience with specialist care, controlling for medication and imaging orders

eTable 8. Association of composite patient experience with specialist care and PCP decisions to direct referrals

This supplemental material has been provided by the authors to give readers additional information about their work.

© 2023 Pany M et al. *JAMA Internal Medicine.*

eMethods

We describe methodological detail essential to understanding our study in the main text. We include additional detail here. (See the main text for a description of the study design, inclusion criteria, and statistical analyses.)

Co-training as a predictor of peer relationships

Overlap in medical training is a formative and salient experience for many trainees. While formal research in this area is lacking, anecdotes about the importance of relationships formed during training abound. Overlap in medical training leads to a substantial number of marriages and lifelong friendships, for instance, and is exemplified by these testimonials from trainees at three different institutions (these are just the top three from a simple google search — there are many more like these):

1. Internal Medicine chief resident (2022-2023) at Emory: "*What is your favorite thing about our program?* 'By far, my favorite thing about our program has definitely been the people! There are so many examples I can think of that exemplify the cameraderie embedded into the culture of our program. In just the last few weeks, as winter storms struck in Texas, I got countless texts and phone calls from thoughtful co-residents checking on me and my family back home. It has been a true pleasure getting to know so many of my co-residents and building friendships with them that will last a lifetime.'"
2. Emergency Medicine resident (class of 2024) at Spectrum Health: "*Why did you choose Spectrum Health for your residency/fellowship?* 'Spectrum Health was on my radar from the beginning since my wife's family lives in the area. I was so excited on interview day when it became apparent that this was my place. It was immediately clear how much the program director cared about residents, how close they all were and what a world class organization Spectrum Health is. The perfect blend of training in an environment that I felt matched my core values, I'm so excited to be at Spectrum Health!'"
3. Emergency Medicine resident (class of 2023) at GW: "*Why did you choose GW for residency?* Many reasons, but especially because it was easy to see on interview day that people at GW really enjoyed being together. It was so apparent they were more than just co-residents and coworkers, but more of friends and family."

© 2023 Pany M et al. *JAMA Internal Medicine.*

The indicator of co-training we construct (details in the main text and below) is a predictor of peer relationships rather than a direct measure of relationships formed. The co-training indicator is necessarily diluted with dyads that did not form relationships, which would tend to underestimate any findings—i.e., bias them toward the null hypothesis. To explore the extent to which PCP-specialist dyads who overlapped in training formed a peer relationship as a result, we identified the subset of PCP-specialist dyads who were medical school classmates or in the same PGY class. We would expect a very high proportion of medical school classmates, co-interns, and co-fellows to know each other well.

Included specialties

We included 13 specialties based on their high referral volume, availability of co-trained PCP-specialist dyads, and representation of a spectrum of specialty types from cognitive to procedural and surgical. The included specialties are allergy and immunology, endocrinology, rheumatology, cardiology, neurology, pulmonology, dermatology, urology, general surgery, obstetrics and gynecology, reproductive endocrinology and infertility (a sub-specialty of obstetrics and gynecology), orthopedic surgery, and neurosurgery.

We pool together specialties and estimate an average effect of the presence of a PCP-specialist co-training relationship on outcomes. The inclusion of specialist fixed effects ensures that any differences in average ratings across specialists, and implicitly their specialties, are controlled for.

Identifying new specialist visits

Because referrals may be used to continue pre-existing patient-specialist relationships and because patient familiarity with specialists may influence their specialist ratings, we restrict our study sample to patient visits new to a specialist. Consistent with the “three-year rule” used in medical billing, we define new specialist visits as those without patient-specialist interaction for at least three years. We implement this definition for study years 2017 to 2019. For study year 2016, due to data availability, we can only ensure that no patient-specialist interaction has happened for at least two years. We include year fixed effects to adjust for the average year-specific effect on outcomes, which removes any potential average differential ratings effect by year due to this difference in how new specialist visits are defined.

Study variables

© 2023 Pany M et al. *JAMA Internal Medicine.*

Physician co-training

We used publicly accessible medical licensing data from the Massachusetts Board of Registration in Medicine (BORIM) to determine co-training status among primary care physician (PCP)-specialist dyads. BORIM's website lists the medical institutions and graduation years of all physicians licensed to practice in Massachusetts, irrespective of state or country of training. We matched physician's National Provider Identifier (NPI) numbers associated with their BORIM record to the NPIs of providers in our study sample. For each PCP in a specialist's referral base—defined as all PCPs working in the physician organizations a specialist received referrals from, irrespective of whether or not particular PCPs had referred to this specialist—we then determined PCP-specialist co-training status. Specifically, we defined as co-training PCP-specialist overlap at the same institution for at least one year during either medical school or post-graduate medical training, irrespective of specialty or stage for the latter (residency or fellowship). This definition of co-training includes potential relationships between students, between residents or fellows, and between residents and fellows, who commonly interact during training, but not between students and residents/fellows. We performed several sensitivity analyses as described in the main text.

Statistical analysis

Estimation of specialist-level variation in patient experience measures

To facilitate interpretation of effect sizes, we simulated the percentile increase in patient experience ratings associated with PCP-specialist co-training in terms of moving from median performance among specialists to the corresponding higher percentile of specialist performance implied by the estimated effect (β_1). Specifically, among undirected referrals, we fitted linear mixed models of composite experience ratings as a function of patient covariates and random effects for the mean rating for each specialist to obtain the estimated standard deviation (SD) of mean ratings across specialists. We then divided the estimated difference in ratings due to co-training (β_1) by the estimated SD of mean ratings across specialists to calculate the effect size in SD units.

Association between co-training and survey response rate

In addition to the secondary analyses described in the main text, we estimated effects of co-training on survey response rates as an additional sensitivity analysis. If patients' altered care experience prompted a different response rate, our estimates could over- or underestimate effects of interest.

© 2023 Pany M et al. *JAMA Internal Medicine.*

eResults

Sample characteristics

The combined sample of directed and undirected referrals with patient experience ratings available included 9,920 specialist visits resulting from PCP-initiated referrals across 13 specialties (306 with co-trainees vs. 9,614 with non-co-trainees) of 8,655 patients (279 vs. 8,422) to 502 specialists (123 vs. 500) initiated by 2,277 PCPs (126 vs. 2,255). *Table S1* shows a breakdown of visits and physicians by specialty type.

Patient experiences with specialist care

Patient experience ratings were routinely collected at two system hospitals. The two hospitals accounted for 29.3% of system-wide specialist visits (n=84,359) resulting from referrals to the 13 included specialties with co-trainees available. Of those visits, 11.7% (n=9,920) had a patient experience survey administered and completed. Based on the reported response rate of 23%, this suggests that about half of the visits in our sample were randomly sampled for survey. Specialist-PCP co-training relationships were not associated with a meaningful difference in the availability of completed patient experience survey data (covariate-adjusted difference: -0.6 percentage points [pp]; 95% CI: -2.6,1.3 pp). In the sample of undirected referrals with patient experience ratings available, 15.9% of PCP-specialist dyads overlapped in training during medical school only, 77.8% during post-graduate training only, and 6.3% during both.

© 2023 Pany M et al. *JAMA Internal Medicine.*

eFigure. Conceptual representation of the study design

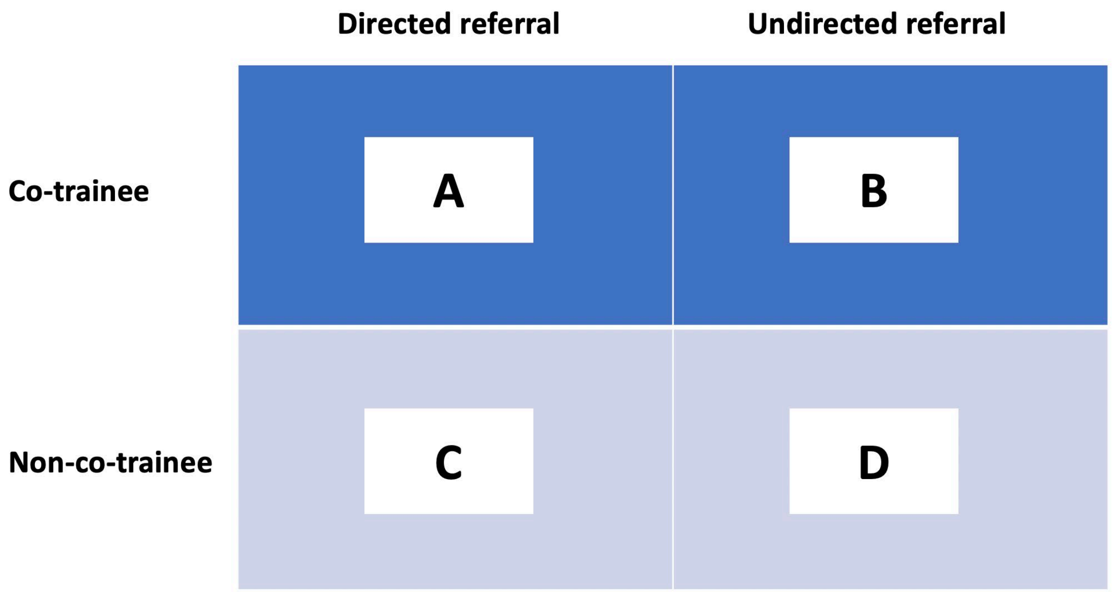

Referrals belong to one of the four categories. We compare patients' experiences with specialist care for referrals from co-trained PCPs compared to those from non-co-trained PCPs both for the pooled sample of directed and undirected referrals (box A + box B vs. C + box D) and for undirected referrals alone (box B vs. D). The extent to which the two estimates differ, if at all, can give insight into the potential influence of PCP preference, patient preference, and PCP-patient communication about the specific specialist, which plausibly occurs only for directed, but not for undirected, referrals.

© 2023 Pany M et al. *JAMA Internal Medicine.*

eTable 1. Combined sample characteristics by specialty

Specialty	Visits, n	Patients, n	PCPs, n	Specialists, n
Allergy and Immunology	307	300	172	22
Cardiology	1,162	799	463	79
Dermatology	1,996	1,905	611	50
Endocrinology	484	473	254	36
General Surgery	567	552	347	31
Neurology	640	623	399	84
Neurosurgery	357	352	287	22
Obstetrics & Gynecology	481	460	233	32
Orthopedic Surgery	2,122	2,027	842	52
Pulmonology	243	240	184	36
Reproductive Endocrinology and Infertility	274	263	236	10
Rheumatology	562	547	338	40
Urology	725	703	374	13

Breakdown of the number of unique visits, patients, referring PCPs, and specialists by specialty included in the sample of directed and undirected referrals with patient experience ratings recorded (as described in the main text).

© 2023 Pany M et al. *JAMA Internal Medicine.*

eTable 2. Non-response to patient experience items

Non-response	Care provider item									
	CP1	CP2	CP3	CP4	CP5	CP6	CP7	CP8	CP9	CP10
No, n	9,844	9,808	9,794	9,577	7,923	9,159	9,753	9,818	9,782	9,704
Yes, n	76	112	126	343	1,997	761	167	102	138	216

Each column contains the count of non-responses and responses to each of the 10 care provider items on the Press Ganey Medical Practice Survey for the sample of directed and undirected referrals with patient experience ratings recorded among included specialties (as described in the main text and Supplement).

© 2023 Pany M et al. *JAMA Internal Medicine.*

eTable 3. **Association between co-training status of PCP-specialist dyad and characteristics of patients seen during visits with specialists, in the sample of undirected referrals with patient experience ratings available**

Variable	Visits with specialists who co-trained with PCP[a] (n=126) †	Visits with specialists who did not co-train with PCP[a] (n=4,232) †	Standardized Mean Difference
**Age, yr**	57.2±11.7	58.1±10.9	-0.084
**Female**, %	66.1	64.1	0.06
**Race and ethnicity,[b] %**			
Asian, Non-Hispanic	5.9	3.6	0.155
Black, Non-Hispanic	6.8	5.0	0.103
Hispanic	0.6	1.2	-0.073
White, Non-Hispanic	80.6	84.8	-0.146
Other	6.0	5.4	0.032
**Preferred language, %**			
English	98.8	97.2	0.122
Spanish	0.8	1.3	-0.061
Other	0.5	1.5	-0.109
**Educational attainment, highest degree, %**			
High school	20.7	18.1	0.086
College	51.9	52.2	-0.006
Graduate school	12.2	16.5	-0.148
Other	15.2	13.2	0.073
**Insurance, %**			
Commercial	57.3	55.4	0.05
Medicare	30.7	32.3	-0.046
Medicaid	7.3	5.8	0.084
Other	4.7	6.5	-0.092
**Elixhauser comorbidity index[c]**	1.8±2.03	2.02±1.87	-0.121

[a] The table shows patient characteristics for the sample that includes undirected referrals with patient experience ratings available. Means or proportions in characteristics, and differences associated with co-training, were estimated and adjusted for specialist and PCP fixed effects by fitting a model for each patient covariate as the dependent variable with an indicator of co-training status and specialist and PCP fixed effects as the independent variables. Plus-minus values are means ± SD. Numbers may not sum to 100% due to rounding.
[b] Race and ethnicity are assessed from electronic health record data, which may not concord with self-reported race and ethnicity. As with other patient covariates, we assessed race and ethnicity to assess balance in patient characteristics associated with co-training relationships.
[c] The Elixhauser comorbidity index summarizes patient comorbidities that are predictive of hospital outcomes, including mortality, and was measured over the 12 months preceding each patient's visit.

© 2023 Pany M et al. *JAMA Internal Medicine.*

eTable 4. **Association between co-training status of PCP-specialist dyad and characteristics of patients seen during visits with specialists, in the sample of system-wide undirected referrals**

Variable	Visits with specialists who co-trained with PCP[a] (n=4,104) †	Visits with specialists who did not co-train with PCP[a] (n=132,970) †	Standardized Mean Difference
**Age, yr**	52.9±14.1	52.7±13.4	0.012
**Female**, %	63.1	63.3	-0.004
**Race and ethnicity,[b] %**			
Asian, Non-Hispanic	4.6	4.7	-0.005
Black, Non-Hispanic	7.8	7.6	0.009
Hispanic	2.9	2.9	0.004
White, Non-Hispanic	75.7	76.1	-0.009
Other	8.9	8.8	0.006
**Preferred language. %**			
English	92.1	91.8	0.013
Spanish	4.5	4.9	-0.018
Other	3.3	3.3	0.001
**Educational attainment, highest degree. %**			
High school	25.4	24.9	0.011
College	40.9	41.8	-0.02
Graduate school	11.9	12.2	-0.009
Other	21.8	21.1	0.019
**Insurance. %**			
Commercial	55.8	55.9	-0.003
Medicare	26	26.2	-0.004
Medicaid	11.5	11.7	-0.008
Other	6.8	6.3	0.023
**Elixhauser comorbidity index[c]**	2.02±2.56	2.09±2.34	-0.03

[a] The table shows patient characteristics for the sample that includes undirected referrals system-wide. Means or proportions in characteristics, and differences associated with co-training, were estimated and adjusted for specialist and PCP fixed effects by fitting a model for each patient covariate as the dependent variable with an indicator of co-training status and specialist and PCP fixed effects as the independent variables. Plus-minus values are means ± SD. Numbers may not sum to 100% due to rounding.

[b] Race and ethnicity are assessed from electronic health record data, which may not concord with self-reported race and ethnicity. As with other patient covariates, we assessed race and ethnicity to assess balance in patient characteristics associated with co-training relationships.

[c] The Elixhauser comorbidity index summarizes patient comorbidities that are predictive of hospital outcomes, including mortality, and was measured over the 12 months preceding each patient's visit.

© 2023 Pany M et al. *JAMA Internal Medicine.*

eTable 5. Decomposition of the co-training effect by overlap in medical school versus post-graduate medical training

**Co-training**	**Estimate**
Medical school	3.7<br>(5.5)
Post-graduate	6.7*<br>(2.8)
PCP fixed effects	Yes
Specialist fixed effects	Yes
Patient covariates	Yes
Observations	4,345
R^2	0.36
Within R^2	0.027

Effect of co-training on composite patient experience with specialist care (in percentage points), decomposed by overlap in medical school versus post-graduate medical training. Coefficient estimates are created by fitting a model with indicators for training overlap in medical school and post-graduate medical training, PCP fixed effects, specialist fixed effects, and patient covariates in the sample of specialist visits resulting from undirected referrals and are shown as percentage points with standard errors in parentheses. Additional details in the main manuscript. * denotes P-value < 0.05.

© 2023 Pany M et al. *JAMA Internal Medicine.*

eTable 6. Decomposition of the co-training effect by full temporal overlap (same-year trainees) versus partial temporal overlap (different-year trainees)

Co-training	Estimate
Full temporal overlap (same-year trainees)	10.1* (4.3)
Partial temporal overlap (different-year trainees)	6.2 (3.4)
PCP fixed effects	Yes
Specialist fixed effects	Yes
Patient covariates	Yes
Observations	4,345
R^2	0.36
Within R^2	0.027

Effect of co-training on composite patient experience with specialist care (in percentage points), decomposed by full temporal overlap (same-year trainees) versus partial temporal overlap (different-year trainees). Coefficient estimates are created by fitting a model with indicators for full and partial temporal overlap, PCP fixed effects, specialist fixed effects, and patient covariates in the sample of specialist visits resulting from undirected referrals and are shown as percentage points with standard errors in parentheses. Additional details in the main manuscript. * denotes P-value < 0.05.

© 2023 Pany M et al. *JAMA Internal Medicine.*

eTable 7. Association between co-training and composite patient experience with specialist care, controlling for medication and imaging orders

**Dependent variable**<br>Composite patient experience	(1)	(2)	(3)	(4)
Co-training	9.0***<br>(1.7)	9.0***<br>(1.7)	7.4*<br>(2.9)	7.4*<br>(2.9)
Control for medication and imaging orders?	Yes	No	Yes	No
Sample	All referrals	All referrals	Undirected referrals	Undirected referrals
PCP fixed effects	Yes	Yes	Yes	Yes
Specialist fixed effects	Yes	Yes	Yes	Yes
Patient covariates	Yes	Yes	Yes	Yes
Observations	9,867	9,867	4,345	4,345
R^2	0.34	0.34	0.36	0.36
Within R^2	0.027	0.026	0.028	0.028

Effect of co-training on composite patient experience with specialist care (in percentage points). Estimates are created by fitting a model with an indicator for co-training, PCP fixed effects, specialist fixed effects, and patient covariates as described in the main manuscript and are shown as percentage points with standard errors in parentheses. Models (1)–(4) vary in whether or not specialist medication and imaging orders are controlled for and whether they were estimated in the sample of all referrals or undirected referrals only. *** denotes P-value < 0.001 and * denotes P-value < 0.05

© 2023 Pany M et al. *JAMA Internal Medicine.*

eTable 8. Association of composite patient experience with specialist care and PCP decisions to direct referrals

**Dependent variable** Composite patient experience	**(1)**	**(2)**
Directed referral	0.1	0.05
	(0.72)	(0.86)
PCP fixed effects	No	Yes
Specialist fixed effects	Yes	Yes
Patient covariates	Yes	Yes
Observations	9,867	9,867
R^2	0.11	0.34
Within R^2	0.026	0.024

Effect of PCPs' decision to direct referrals on composite patient experience with specialist care (in percentage points). Coefficient estimates are created by fitting a model with indicators for directed referrals, PCP fixed effects, specialist fixed effects, and patient covariates in the sample of specialist visits resulting from directed and undirected referrals and are shown as percentage points with standard errors in parentheses. Models (1) and (2) vary in the covariates included. Across both models, the impact of directing a referral on composite patient experience with specialist care was small and not statistically significant, suggesting that PCP-patient communication or patient requests for particular specialists did not meaningfully affect patient experience with specialist care.

© 2023 Pany M et al. *JAMA Internal Medicine.*
