# Supplementary material for: Physician-Peer Relationships and Patient Experiences With Specialist Care
Source: JAMA Intern Med. 2023 Jan 3:e226007. Online ahead of print. doi: 10.1001/jamainternmed.2022.6007 (PMC9857606; doi:10.1001/jamainternmed.2022.6007)
Supplement: Supplement 2. — Data sharing Statement [file jamainternmed-e226007-s002.pdf]

Data Sharing Statement

Pany. Physician-Peer Relationships and Patient Experiences With Specialist Care. *JAMA Intern Med*. Published January 03, 2023. doi:10.1001/jamainternmed.2022.6007

Data
Data available: No

Additional Information
Explanation for why data not available: The data is confidential electronic health record data and as such cannot be made available.
